# *Acinetobacter baumannii* response to cefiderocol challenge in human urine

**DOI:** 10.1038/s41598-022-12829-7

**Published:** 2022-05-24

**Authors:** Brent Nishimura, Jenny Escalante, Marisel R. Tuttobene, Tomás Subils, Vyanka Mezcord, Camila Pimentel, Nardin Georgeos, Fernando Pasteran, Cecilia Rodriguez, Rodrigo Sieira, Luis A. Actis, Marcelo E. Tolmasky, Robert A. Bonomo, María Soledad Ramirez

**Affiliations:** 1 Center for Applied Biotechnology Studies, Department of Biological Science, College of Natural Sciences and Mathematics, California State University Fullerton, 800 N State College Blvd, Fullerton, CA 92831 USA; 2 Área Biología Molecular, Facultad de Ciencias Bioquímicas y Farmacéuticas, Universidad Nacional de Rosario, Rosario, Argentina; 3 Instituto de Biología Molecular y Celular de Rosario (IBR, CONICET-UNR), Rosario, Argentina; 4 Instituto de Procesos Biotecnológicos y Químicos de Rosario (IPROBYQ, CONICET-UNR), Rosario, Argentina; 5 National Regional Reference Laboratory for Antimicrobial Resistance (NRL), Servicio Antimicrobianos, Instituto Nacional de Enfermedades Infecciosas, ANLIS Dr. Carlos G. Malbrán, Buenos Aires, Argentina; 6 Centro de Referencia Para Lactobacilos (CERELA), CONICET, Tucumán, Argentina; 7 Instituto Leloir–IIBBA CONICET, Buenos Aires, Argentina; 8 Department of Microbiology, Miami University, Oxford, USA; 9 Research Service and GRECC, Louis Stokes Cleveland Department of Veterans Affairs Medical Center, Cleveland, OH USA; 10 Departments of Medicine, Pharmacology, Molecular Biology and Microbiology, Biochemistry, Proteomics and Bioinformatics, Case Western Reserve University School of Medicine, Cleveland, OH USA; 11 CWRU-Cleveland VAMC Center for Antimicrobial Resistance and Epidemiology (Case VA CARES), Cleveland, OH USA

**Keywords:** Microbiology, Antimicrobials, Clinical microbiology

## Abstract

Cefiderocol (CFDC) is a novel chlorocatechol-substituted siderophore antibiotic approved to treat complicated urinary tract infections (cUTI) and hospital-acquired and ventilator-acquired pneumonia (HAP/VAP). Previous work determined that albumin-rich human fluids increase the minimum inhibitory concentration (MICs) of *Acinetobacter baumannii* against CFDC and reduce the expression of genes related to iron uptake systems. This latter effect may contribute to the need for higher concentrations of CFDC to inhibit growth. The presence of human urine (HU), which contains low albumin concentrations, did not modify MIC values of two carbapenem-resistant *A. baumannii*. Levels of resistance to CFDC were not modified by HU in strain AMA40 but were reduced in strain AB5075. Expanding the studies to other carbapenem-resistant *A. baumannii* isolates showed that the presence of HU resulted in unmodified or reduced MIC of CDFC values. The expression of *piuA, pirA, bauA*, and *bfnH* determined by qRT-PCR was enhanced in *A. baumannii* AMA40 and AB5075 by the presence of HU in the culture medium. All four tested genes code for functions related to recognition and transport of ferric-siderophore complexes. The effect of HU on expression of *pbp1*, *pbp3, bla*_OXA-51-like_, *bla*_ADC_, and *bla*_NDM-1_, genes associated with resistance to β-lactams, as well as genes coding for efflux pumps and porins was variable, showing dependence with the strain analyzed. We conclude that the lack of significant concentrations of albumin and free iron in HU makes this fluid behave differently from others we tested. Unlike other albumin rich fluids, the presence of HU does not impact the antibacterial activity of CFDC when tested against *A. baumannii.*

## Introduction

*Acinetobacter baumannii* is a versatile pathogen responsible for hospital-acquired and ventilator-associated pneumonia (HAP/VAP), bacteremia, as well as complicated urinary tract (cUTI) and wound infections, especially in intensive care unit patients^1–4^. The World Health Organization (WHO) and the Centers for Disease Control and Prevention (CDC) identified *A. baumannii* as a “top priority” for the research and development of new antimicrobial therapies^5, 6^. Despite *A. baumannii* being the causative agent of common and catheter-associated cUTIs, and these infections accounting for about 20% of *A. baumannii* clinical isolates^7, 8^. The majority of research on this bacterium’s pathogenicity has focused on pneumonia and bloodstream infections to the detriment of the mechanisms that lead to infections in the human urinary tract*.*

Changes in gene expression when *A. baumannii* is exposed to human blood or serum has attracted significant interest. The presence of these fluids creates a hostile environment by limiting nutrients, such as iron, or actively damaging the bacterial cells through immunity mechanisms^9, 10^. For example, when *A. baumannii* is grown in human serum albumin (HSA), this pathogen responds by modifying the expression of critical genes^10–16^, such as those related to iron uptake systems and resistance to β-lactams^17^. Another effect that was observed in these conditions is an increase in the minimum inhibitory concentrations (MICs) to cefiderocol (CFDC), a novel antibiotic approved by Food and Drug Administration (https://www.accessdata.fda.gov/drugsatfda_docs/label/2019/209445s000lbl.pd). Since CFDC is a cephalosporin antibiotic conjugated to a siderophore, transport into the bacterial periplasm to inactivate penicillin binding proteins (PBPs) depends on receptors regulated by the iron concentration in the milieu. An attractive hypothesis to explain these results is that when *A. baumannii* grows in the presence of HSA-containing human fluids, there is down-regulation the expression of iron uptake system genes, reducing the ability of CDFC to reach the cell’s PBPs^17^. Since albumin is absent or present in trace concentrations in normal HU, we hypothesize that CFDC efficacy would not be reduced when *A. baumannii* is exposed to HU.

Previous studies showed that CFDC is safe and effective for the treatment of cUTI in patients at risk of multidrug-resistant (MDR) Gram-negative infections, including secondary bacteremia^18, 19^. In addition, CFDC did not demonstrate inferiority to imipenem-cilastatin^19^. However, patients with carbapenem-resistant infections were not included in this study. In this work, we evaluate the effect of HU on representative carbapenem-resistant *A. baumannii* (CRAB) strains with different genetic backgrounds focusing on changes in CFDC MICs and expression levels of iron uptake TonB-dependent receptors and β-lactam resistance genes.

## Results and discussion

### cUTIs and *A. baumannii*: a review of the clinical problem

In order to provide clinical context to this work, we focused our attention herein on *A. baumannii* infections in Argentina as a representative country to illustrate the magnitude of the medical problem.

A review of data analysis indicated that the incidence of CRAB in Argentina pre-COVID-19 was 8 in 10,000 patients, while after the arrival of COVID-19, the incidence in 2020 was 32 in 10,000. In addition, three out of the 32 infections were *A. baumannii* recovered from urinary sites while the pneumonia and bloodstream infections represent 55% and 25% respectively (Personal communication provided by the National Reference Laboratory and the National Commission for the Control of Antimicrobial Resistance, National Ministry of Health). On the other hand, during the period January 2021–2022, a total of 1758 strains isolated from urinary sites were recorded at the main adult hospital in the province of Tucumán (northern Argentina). Nineteen of these isolates (1.08%), corresponded to *A. baumannii*, of which 17 isolates (89.47%) were CRAB. While 86 *A. baumannii* strains (16.86%) were recovered from pneumonia infection out of a total of 510 isolates, of which 84 strains (97.67%) corresponded to CRAB. In bloodstream infections, out of a total of 502 isolates, 25 corresponded to *A. baumannii* (4.98%), of which 20 isolates (80%) were CRAB.

### Modulation of the expression levels of genes associated with iron-uptake systems in the presence of HU

Iron uptake systems are expressed in early stages of *A. baumannii* infections under iron-restricted conditions^20^. The iron transporters *bauA* and *bfnH*, which are coupled to a TonB energy transduction system needed to translocate the siderophore-iron complex into the periplasm, were characterized in *A. baumannii*^21^. Jacobs et al.^22^ reported that the expression of *bauA* and *bfnH* were enhanced during an infection caused by *A. baumannii*. In addition, these genes were identified as vaccine candidates using reverse vaccinology^23^. Moreover, *piuA* and *pirA* encoded for two novel TonB-dependent receptors of *A. baumannii,* which are associated with iron uptake^24^.

To study *A. baumannii* response to HU (fluids with traces or undetectable level of HSA) and its possible effect on the expression level of iron transporters genes (*piuA, pirA, bauA*, and *bfnH)*, we performed quantitative RT-PCR (qRT-PCR) assays. Total RNA extracted from two representative CRAB model strains (AB5075 and AMA40) cultured in iron-depleted cation adjusted Mueller Hinton broth (CAMHB), or CAMHB supplemented with 50% HU was used.

**Figure 1:**
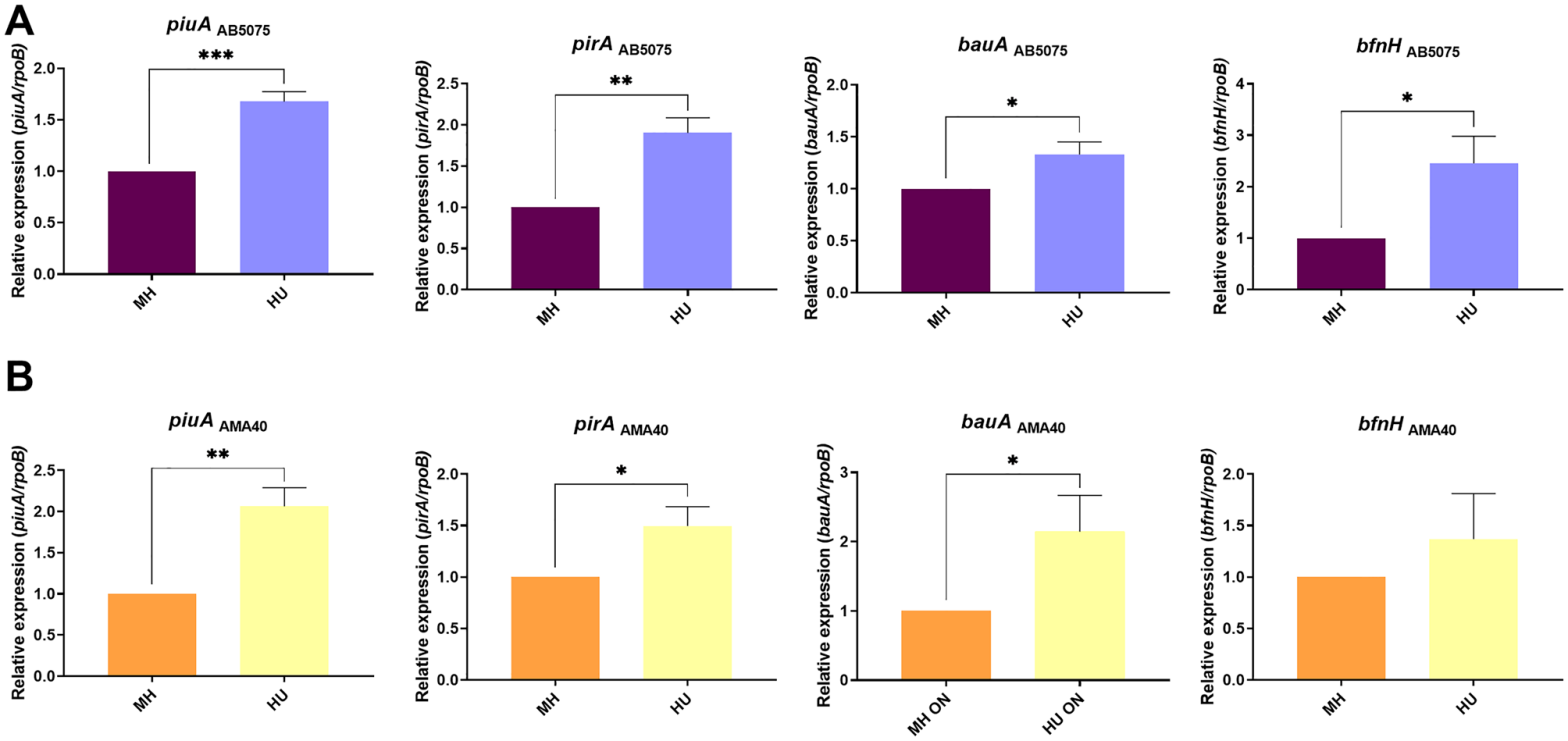
Genetic analysis of iron uptake genes of AB5075 (**A**) and AMA40 (**B**) *A. baumannii* strains. qRT-PCR of genes associated with iron uptake, *pirA, piuA, bauA* and *bfnH* expressed in cation adjusted Mueller Hinton or cation adjusted Mueller Hinton supplemented with 50% HU. Fold changes were calculated using double ΔCt analysis. At least three independent samples were used, and four technical replicates were performed from each sample. The cation adjusted Mueller Hinton was used as reference. Statistical significance (*p* < 0.05) was determined by ANOVA followed by Tukey’s multiple-comparison test, one asterisks: *p* < 0.05; two asterisks: *p* < 0.01 and three asterisks: *p* < 0.001.

The expression of *piuA, pirA, bauA*, and *bfnH* was significantly enhanced in both strains when cultured in the presence of HU (Fig. 1A,B). Previous results showed that when the AB5075, AMA16 and AB0057 CRAB strains were exposed to pure 3.5% HSA, or 4% HPF or 100% HS, fluids containing HSA, the transcriptional expression levels of these genes were down regulated for most of the conditions and strains studied^17^. The total Fe concentration was determined in 3.5% HSA and HU, achieving 72 µM; no additional iron was detected, respectively (as determined by a colorimetric iron assay kit, Sigma-Aldrich, MO, USA). Moreover, the total Fe concentration in HPF is about 130 µM^12^. Therefore, we conclude that in HU, the expression levels of genes related to the acquisition of iron are upregulated (Fig. 1), in contrast to the effect observed with fluids containing high content of HSA.

### Changes in the expression levels of β-lactam resistance genes in the presence of HU

**Figure 2:**
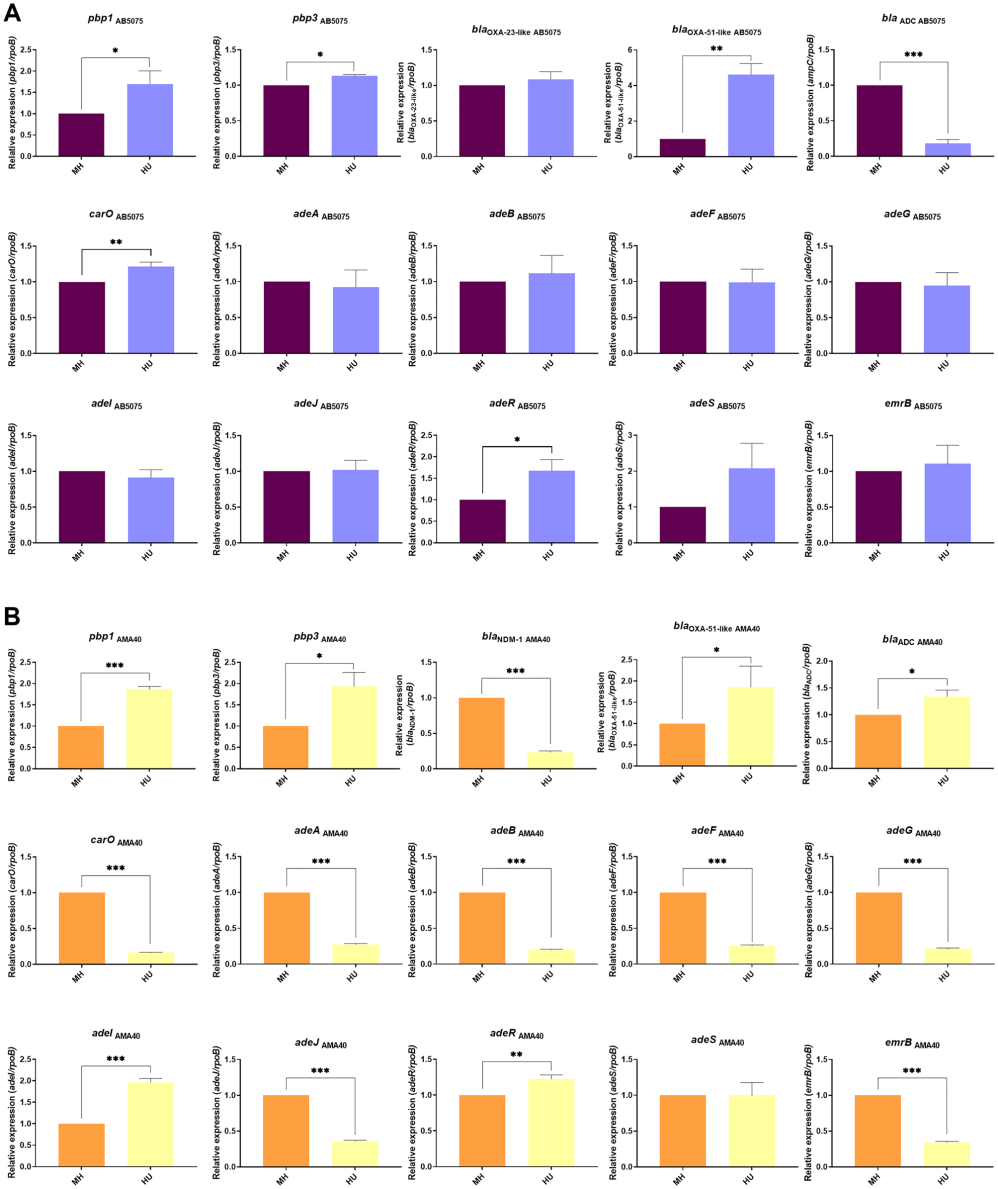
Genetic analysis of β-lactamase and PBP genes of AB5075 (**A**) and AMA40 (**B**) *A. baumannii* strains. qRT-PCR of genes associated with β-lactams resistance, efflux pump, and porins expressed in cation adjusted Mueller Hinton, cation adjusted Mueller Hinton supplemented with 50% HU. Fold changes were calculated using double ΔCt analysis. At least three independent samples were used. LB was used as the reference condition. Statistical significance (*p* < 0.05) was determined by ANOVA followed by Tukey’s multiple-comparison test, one asterisks: *p* < 0.05; two asterisks: *p* < 0.01, and three asterisks: *p* < 0.001.

To further explore if changes in the expression of genes associated with β-lactam resistance are affected by HU in the selected CRAB strains, qRT-PCR was used to evaluate the expression of *pbp1*, *pbp3, bla*_OXA-23_, *bla*_OXA-51-like_, *bla*_ADC_ and *bla*_NDM-1_ in AB5075 and AMA40 exposed to HU (Fig. 2).

This transcriptional analysis revealed an up-regulation in the expression levels of *pbp1*, *pbp3,* and *bla*_OXA-51-like_ in both strains analyzed. In contrast, a decrease in the level of expression of *bla*_ADC_ in AB5075, and *bla*_NDM-1_ in AMA40 clinical isolate was observed. Changes were not observed for *bla*_OXA-23_ in AB5075, while an increase in *bla*_ADC_ expression was seen for AMA40 (Fig. 2).

To further determine if changes at the transcriptional levels of genes codifying for efflux pumps such as AdeABC, AdeFGH, AdeIJK, and EmrAB, or the CarO porin could contribute to the altered CFDC resistance, qRT-PCR experiments were also carried out. The results showed an increase in the expression levels of *carO* in the AB5075 strain, while there was a decrease in the expression of this gene in the AMA40 strain (Fig. 2).

The analysis efflux pump associated genes varies according to the strain and gene analyzed (Fig. 2). The expression level of seven efflux pumps genes was significantly down-regulated in AMA40 under HU (Fig. 2), while in AB5075 most of the genes did not show a significant change in the expression when exposed to HU (Fig. 2). These results suggest that strains with different carbapenem resistance mechanisms could respond differently to modifications of external conditions.

### RNA sequencing analysis of two carbapenem-resistant *A. baumannii* strains expose to HU

The RNA-seq analysis of *A. baumannii* strains AB5075 and AMA40 revealed that HU significantly affect the expression of 264 and 455 coding genes, respectively (FDR < 0.05 and log2 fold change > 1). In AB5075 strain, 148 genes were up-regulated and 116 were downregulated in the presence of HU. Furthermore, in AMA40 strain, 262 genes were up-regulated and 193 were downregulated in the presence of HU (Table S1).

**Figure 3:**
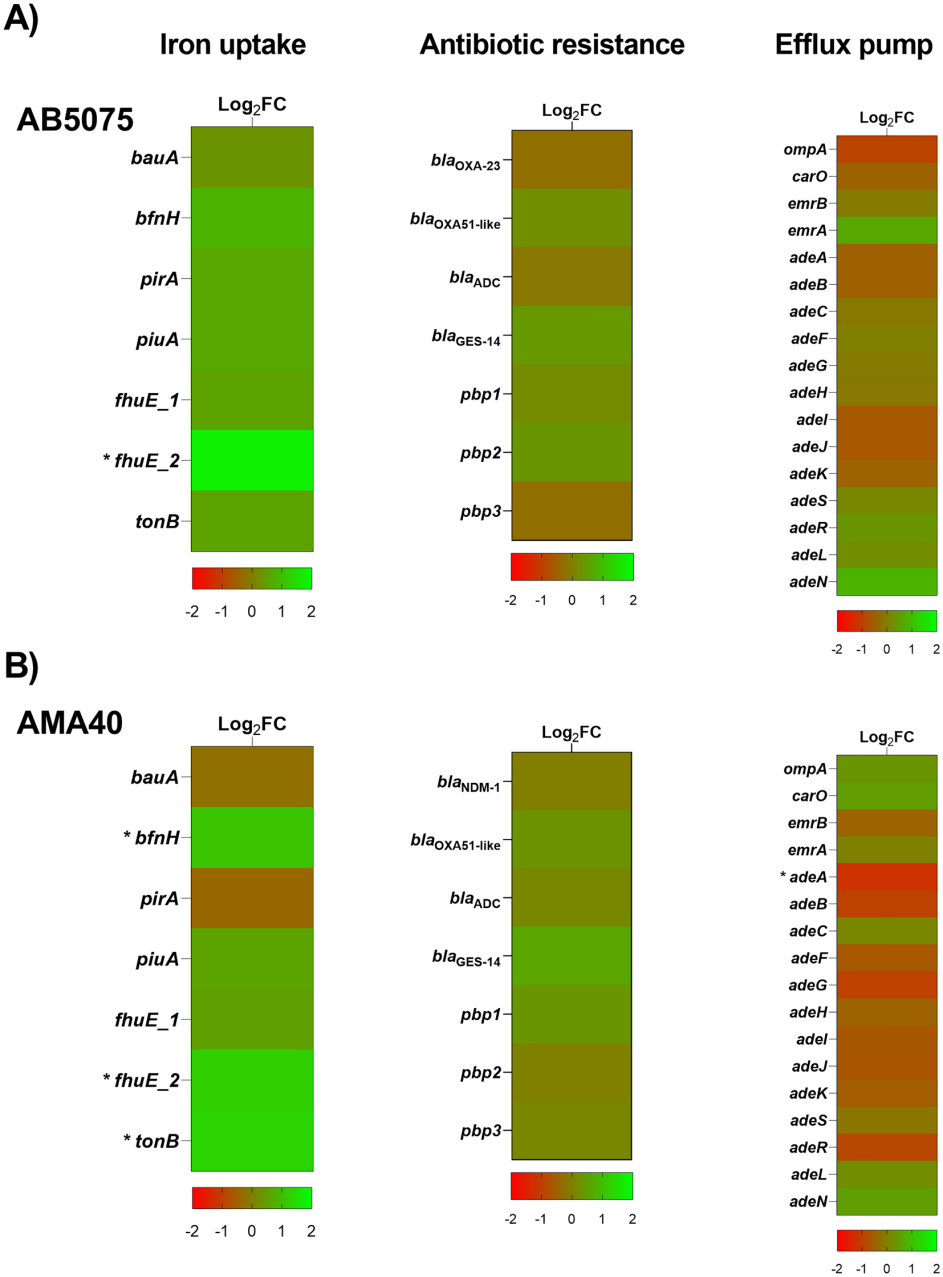
Genetic analysis of iron uptake, antibiotic resistance and efflux pump coding genes by RNA-seq. Heatmap outlining the differential expression of genes associated with iron uptake, antibiotic resistance and efflux pump in presence of HU in *A. baumannii* AB5075 (**A**) and AMA40 (**B**) strains. The asterisks represent the differentially expressed genes (DEGs) (adjusted p < 0.05 with log2fold change > 1).

In the present study, our analysis was focused on genes related to iron uptake, β-lactam resistance, and efflux pumps (Fig. 3 and Table S2). Results obtained confirmed the results determined by qRT-PCR. In addition, the RNA-seq study shows that the expression of *fhuE_2* was significantly up-regulated when both strains were exposed to HU. The expression levels of *bfnH* and *tonB* were also increased under HU in AMA40. In addition, the expression of *adeA* was significantly down-regulated in AMA40 (Fig. 3 and Table S2).

### CRAB strains susceptibility to CFDC in the presence of HU

**Figure 4:**
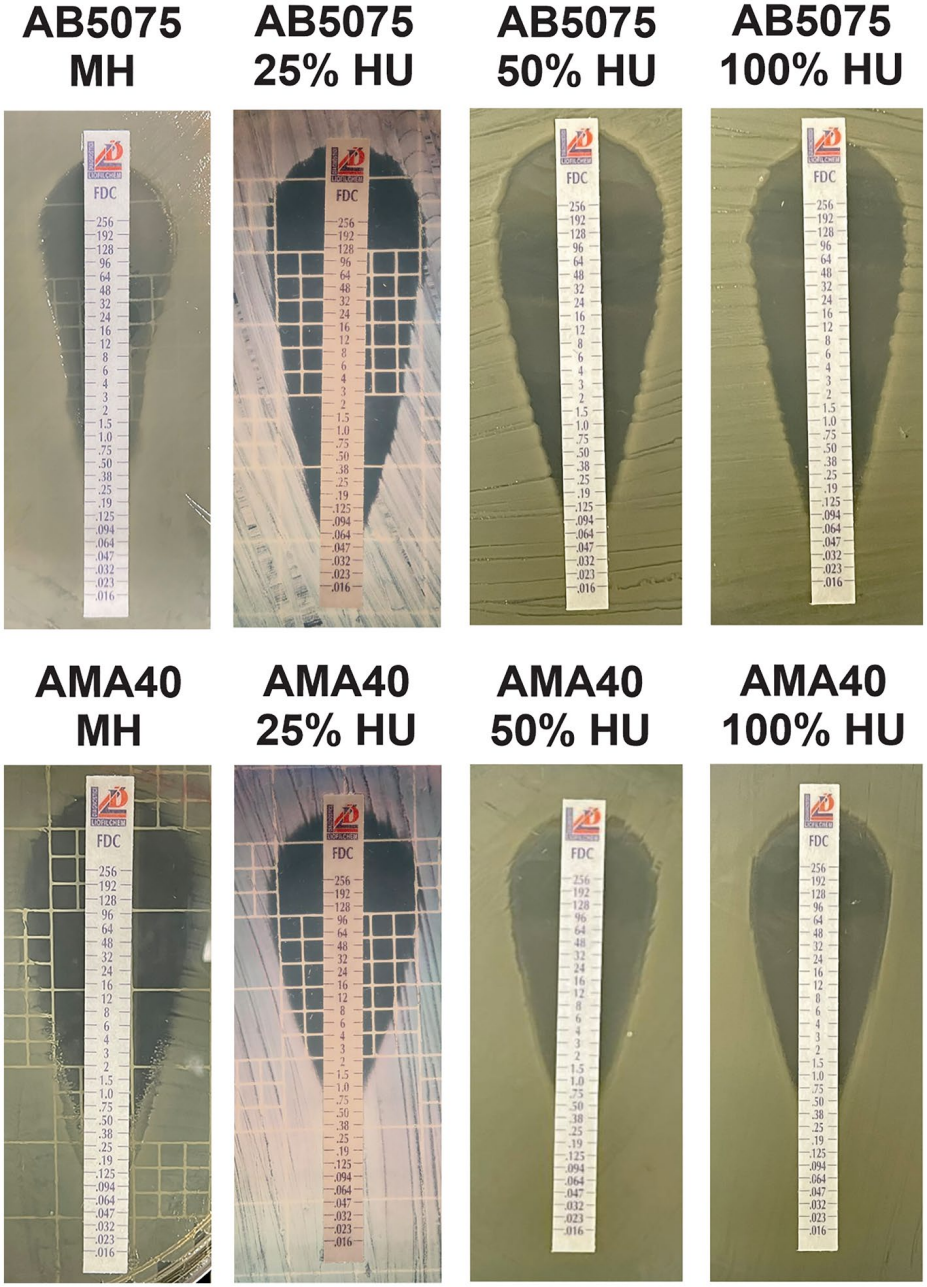
Effect of HU on the antimicrobial susceptibility of *A. baumannii* strains AB5075 and AMA40 strains grew in MH broth, MH broth plus 25, 50 or 100% HU were used to performed cefiderocol (CFDC) susceptibility. Minimum inhibitory concentration (MIC) on cation adjusted Mueller Hinton agar was performed by MTS (Liofilchem S.r.l., Italy) following manufacturer’s recommendations.

Based on these results, AB5075 and AMA40 cells grown in 100% HU or CAMHB that was supplemented with 0%, 25%, or 50% HU were used to determine CFDC MICs. Differences were not observed in CFDC MICs for AMA40 under the tested conditions. However, a twofold decrease in CFDC MIC was observed when AB5075 cells were grown in the presence of HU (Fig. 4 and Table 1). As the level of expression of *bla*_NDM-1_ was down-regulated when exposed to HU in the strain AMA40, MICs for imipenem (IMP) and meropenem (MEM) were performed. This analysis showed that MICs differences for MER was not seen; and a slight decrease (onefold decrease) was seen when testing susceptibility to IMI in presence of HU. We attribute this to the decrease in NDM-1 expression observed when AMA40 was grown in HU.

**Table 1:** Minimal Inhibitory Concentrations (MICs) of cefiderocol (CFDC) for AB5075 and AMA40 Carbapenem-resistant *Acinetobacter baumanii* representative strains performed using CFDC MTS strips (Liofilchem S.r.l., Italy) on Iron-depleted CAMHA (Cation Adjusted Mueller Hinton Agar).

MICs E-test (mg/L)				
Strain	MH	MH 25% HU	MH 50% HU	100% HU
AB5075	0.5 (S)	0.094 (S)	0.094 (S)	0.125 (S)
AMA40	0.5 (S)	1.5 (S)	0.75 (S)	0.5 (S)

HU: Human Urine. *A. baumannii* cells were cultured in MH, MH supplemented with HU 25%, 50% or 100% HU.

To expand our observations to other CRAB strains with different genetic backgrounds and carbapenem resistance mechanisms, CFDC MICs of 11 CRAB selected strains grown in CAMHB or 50% HU were determined. The model strains AB0057 and AYE were included among the tested strains. A previously observed increase in the AB0057 MIC to CDFC when this isolate was cultured in presence of HPF was noted^17^. However, in this case, changes in MIC were not observed under HU conditions (Fig. S1 and Table S3).

Taken together we conclude that the presence of HU resulted in either an increase in susceptibility or no changes in the CFDC MIC in 4 and 6 out of the 11 tested strains, respectively. Lastly, a broader time span was employed to study the adaptive response of the *A. baumannii* to CFDC. To this end, MICs were performed with 48- and 72-h exposure to HU. Results show that CFDC MIC was twofold increase when AB5075 was expose to HU for 72 h, while was onefold increase in AMA40 (Table S4). These results suggest that there is not a clear adaptive response of *A. baumannii* to CFDC when exposed to HU in the condition tested.

## Concluding remarks

UTIs are one of the most common bacterial infections caused by MDR Gram-negative bacteria. Among *A. baumannii* clinical isolates, one in five are from urinary sites according to a retrospective analysis of all *Acinetobacter* isolates identified in the BJC Healthcare System (BJC) from January 2007 through August 2017. *Acinetobacter* spp. pathogenicity has been extensively studied in pneumonia and bloodstream infection models. Past studies showed that the potency of CFDC is reduced when bacteria are exposed to human fluids that contain HSA, presumably due to iron bound to the protein.

Here we tested the effect of another human fluid, urine, which has none or negligible amounts of HSA or free iron. Interestingly, the MIC values of CFDC were not significantly modified by the presence of HU in the testing conditions used. An attractive hypothesis to explain the lack of effect in this bodily fluid as opposed to HSA and HPF is that the regulatory factor in the latter fluids is the iron attached HSA. The implications of these observations may define the more appropriate use of this and other antibiotics to treat MDR infections. Further studies are underway to explore this hypothesis.

## Materials and methods

### Bacterial strains

The model carbapenem-resistant *A. baumannii* strains AB5075 (*bla*_OXA-23_ and *bla*_OXA-51_)^12, 25^ and the carbapenem-resistant clinical AMA40 (*bla*_NDM-1_ and *bla*_OXA-51_) strain^26, 27^ were used in this work. In addition, a total of nine clinical isolates of carbapenem-resistant *A. baumannii* (CRAB) were included in this study. Among these strains, AB0057 isolated in the Walter Reed Army Medical Center^28^ and harboring *bla*_TEM-1_, *bla*_OXA-23_ and *bla*_ADC_ genes was included.

### RNA extraction, quantitative reverse transcription polymerase chain reaction (qRT-PCR), sequencing, and transcriptomic analysis

Overnight cultures of *A. baumannii* strains were diluted 1:10 in fresh iron depleted cation adjusted Mueller Hinton or cation adjusted Mueller Hinton supplemented with 50% of human urine (HU) and incubated with agitation for 18 h at 37 °C. HU was obtained from a certified vendor (Innovative Research Inc, Novi, MI, USA). RNA was extracted from each strain using the Direct-zol RNA Kit (Zymo Research, Irvine, CA, USA) following manufacturer’s instructions. Total RNA extractions were performed in three biological replicates for each condition. The extracted and DNase treated RNA was used to synthesize cDNA using the manufacturer’s protocol provided within the iScriptTM Reverse Transcription Supermix for qPCR (Bio-Rad, Hercules, CA, USA). The cDNA concentrations were adjusted to 50 ng/µL and qPCR was conducted using the qPCRBIO SyGreen Blue Mix Lo-ROX following manufacturer’s protocol (PCR Biosystems,Wayne, PA, USA). At least three biological replicates of cDNA were used in triplets and were run using the CFX96 TouchTM Real-Time PCR Detection System (Bio-Rad, Hercules, CA, USA). Transcriptional levels of each sample were normalized to the transcriptional level of *rpoB*. The relative quantification of gene expression was performed using the comparative threshold method 2^−ΔΔCt^. The ratios obtained after normalization were expressed as folds of change compared with cDNA samples isolated from bacteria cultures on LB. Asterisks indicate significant differences as determined by ANOVA followed by Tukey’s multiple comparison test (p < 0.05), using GraphPad Prism (GraphPad software, San Diego, CA, USA).

RNA sequencing was outsourced to Novegene (Novogene Corporation, Sacramento,CA, USA), where the RNA-seq library preparation (Illumina, San Diego, CA, USA) and HiSeq 2500 paired-end 150 bp sequencing of three independent biological replicates in the presence or absence of HU was performed for AB5075 and AMA40 strains. Trimming of low-quality bases at the ends of the reads to a minimum length of 100 bp and removal of Illumina adaptor sequences was performed using Trimmomatic^29^, yielding an average of 7.6 million paired reads per sample. FastQC http://www.bioinformatics.babraham.ac.uk/projects/fastqc/) (January 2019) was used to assess the quality of the reads before and after trimming. Burrows–Wheeler Alignment software (BWA) was used to align the RNA-seq reads to the genome of *Acinetobacter baumannii*^30^. The alignments were visualized using the Integrated Genome Viewer software^31^. FeatureCounts (REF) was used to calculate the read counts per gene^32^, and differential expression analysis was performed using DEseq^33^. Features exhibiting FDR < 0.05 and log2fold change > 1 were considered statistically significant. Both RNA-seq data were deposited in the Gene Expression Omnibus (GEO) database under the accession number GSE201259.

### Antimicrobial susceptibility testing

Antibiotic susceptibility assays were performed following the procedures recommended by the Clinical and Laboratory Standards Institute (CLSI)^34^. After OD adjustment, 100 µL of cells grown in fresh cation adjusted Mueller Hinton broth (CAMHB) or CAMHB supplemented with 25, 50 or 100% HU were inoculated on CAMHB agar plates as previously described^12^. Antimicrobial commercial E-strips (Liofilchem S.r.l., Roseto degli Abruzzi, Italy) for cefiderocol (CFDC) was used. Mueller–Hinton agar plates were incubated at 37 °C for 18 h. CLSI breakpoints were used for interpretation^34^. *E. coli* ATCC 25922 was used for quality control purposes.

### Determination of total iron concentration

Total iron concentration of HU and LB supplemented with 3.5% HSA was determined using the Iron Assay Kit (Sigma-Aldrich) following the manufacturer’s recommendations. In addition, the iron content was also measured in LB supplemented with 4% HPF confirming previous published results^12^.

## Supplementary Information

Supplementary Figure S1.

Supplementary Table S1.

Supplementary Table S2.

Supplementary Table S3.

Supplementary Table S4.

## Data Availability

The datasets used and/or analyzed during the current study available from the corresponding author on reasonable request. The datasets generated and analyzed during the current study are available in the Gene Expression Omnibus (GEO) database repository under the accession number GSE201259.
