# Supplementary material for: Acinetobacter baumannii response to cefiderocol challenge in human urine
Source: Sci Rep. 2022 May 24;12:8763. doi: 10.1038/s41598-022-12829-7 (PMC9128776; doi:10.1038/s41598-022-12829-7)

**Figure S1:** Effect of human urine (HU) on the antimicrobial susceptibility of *A. baumannii* strains. AB0057 and 8 strains grew in MH broth or MH broth plus 25, 50 or 100 % HU, were used to performed CFDC susceptibility following CLSI recommendations.


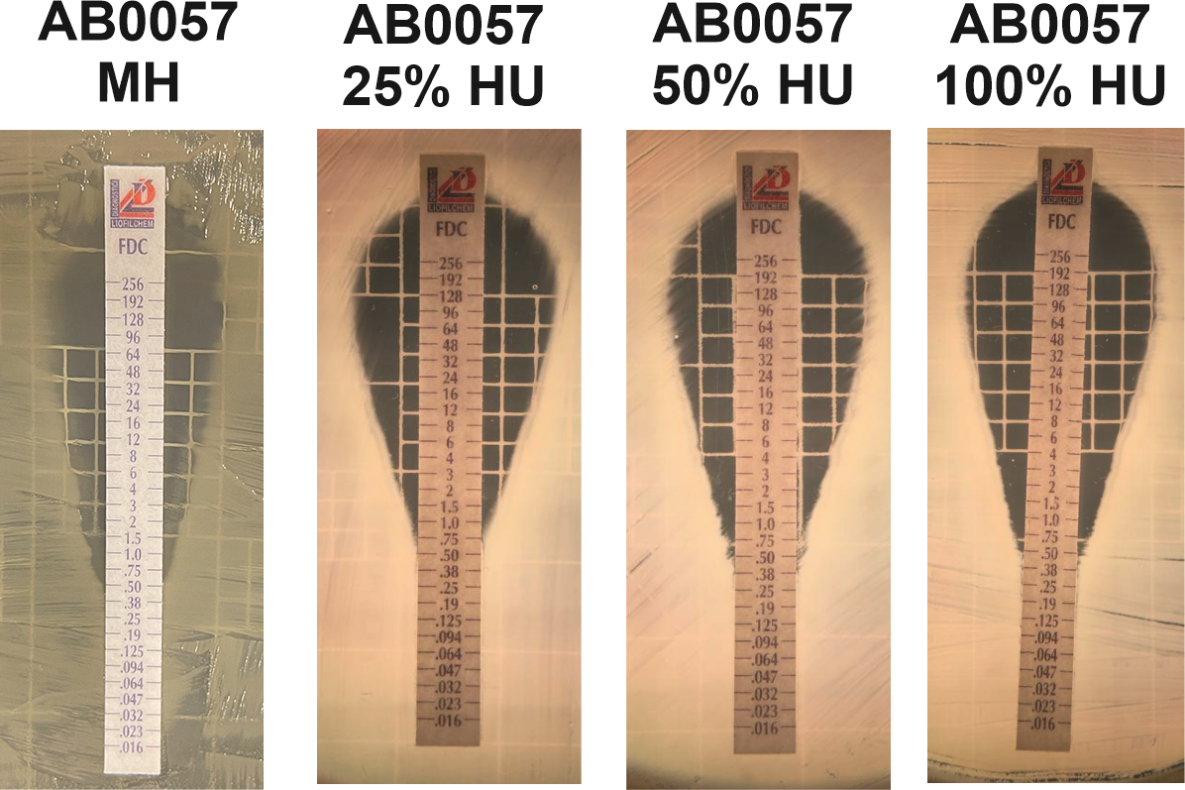

Supplement: Supplementary file 1 — Supplementary Figure S1. [file 41598_2022_12829_MOESM1_ESM.docx]
