# Supplementary material for: Acinetobacter baumannii response to cefiderocol challenge in human urine
Source: Sci Rep. 2022 May 24;12:8763. doi: 10.1038/s41598-022-12829-7 (PMC9128776; doi:10.1038/s41598-022-12829-7)
Supplement: Supplementary file 3 — Supplementary Table S2. [file 41598_2022_12829_MOESM3_ESM.docx]

**Supplementary Table S2:** Transcriptome analysis of *A. baumannii* strain AB5075 versus AB5075 50% HU, and AMA40 versus AMA40 50% HU showing the genes associated with iron uptake, antibiotic resistance and efflux pump.

| ***Acinetobacter baumannii* AB5075** | | | | |
| --- | --- | --- | --- | --- |
| **Iron uptake** | **Gene name** | **Log_2_FC** | **FDR (P-adjusted)** | **Gene Function** |
| ABUW_1177 | *bauA* | 0,2902 | 3,56E-02 | bauA, TonB-dependent ferric acinetobactin receptor |
| ABUW_2182 | *bfnH* | 0,7990 | 5,02E-11 | TonB-dependent receptor |
| ABUW_3403 | *pirA* | 0,6145 | 5,96E-06 | TonB-dependent siderophore receptor |
| ABUW_2916 | *piuA* | 0,6088 | 7,43E-06 | TonB-dependent siderophore receptor |
| ABUW_1655 | *fhuE_1* | 0,5470 | 1,12E-03 | TonB-dependent siderophore receptor |
| ABUW_2165 | *fhuE_2* | 1,7680 | 3,38E-43 | TonB-dependent siderophore receptor |
| ABUW_3426 | *tonB* | 0,5536 | 1,65E-05 | energy transducer TonB |
| **Antibiotic resistance** | | | | |
| ABUW_0563 | *bla*_OXA23_ | -0,2727 | 2,31E-02 | carbapenem-hydrolyzing class D beta-lactamase |
| ABUW_2300 | *bla*_OXA51-like_ | 0,2185 | 4,21E-01 | OXA-51 family carbapenem-hydrolyzing class D |
| ABUW_1194 | *bla*_ADC_ | -0,1414 | 4,97E-01 | class C extended-spectrum beta-lactamase ADC-11 |
| ABUW_1070 | *bla*_GES-14_ | 0,3782 | 1,61E-02 | D-alanyl-D-alanine carboxypeptidase PBP6B |
| ABUW_1358 | *pbp1* | 0,1657 | 2,89E-01 | mrcB, penicillin-binding protein 1B |
| ABUW_2876 | *pbp2* | 0,3466 | 2,03E-02 | mrdA, penicillin-binding protein 2 |
| ABUW_0283 | *pbp3* | -0,2610 | 6,81E-02 | ftsI, penicillin-binding protein PBP3 |
| **Efflux pumps** | | | | |
| ABUW_3045 | *ompA* | -0,9343 | 8,08E-17 | OmpA family protein |
| ABUW_1015 | *carO* | -0,4662 | 1,84E-04 | carO, ornithine uptake porin CarO type 3 |
| ABUW_3019 | *emrB* | -0,0965 | 6,45E-01 | DHA2 family efflux MFS transporter permease |
| ABUW_3020 | *emrA* | 0,6056 | 1,59E-05 | EmrA/EmrK family multidrug efflux transporter |
| ABUW_1974 | *adeA* | -0,4816 | 2,02E-03 | adeA, multidrug efflux RND transporter periplasmic |
| ABUW_1975 | *adeB* | -0,5040 | 1,26E-04 | adeB, multidrug efflux RND transporter permease |
| ABUW_1976 | *adeC* | -0,1326 | 6,25E-01 | adeC, multidrug efflux RND transporter outer membrane |
| ABUW_1336 | *adeF* | -0,0092 | 9,82E-01 | adeF, multidrug efflux RND transporter periplasmic |
| ABUW_1335 | *adeG* | -0,0772 | 7,99E-01 | adeG, multidrug efflux RND transporter permease |
| ABUW_1334 | *adeH* | -0,1371 | 7,15E-01 | adeH, multidrug efflux RND transporter outer membrane |
| ABUW_0844 | *adeI* | -0,6092 | 2,91E-05 | adeI, multidrug efflux RND transporter periplasmic |
| ABUW_0843 | *adeJ* | -0,6089 | 1,59E-06 | adeJ, multidrug efflux RND transporter permease |
| ABUW_0842 | *adeK* | -0,4597 | 1,40E-04 | adeK, multidrug efflux RND transporter outer membrane |
| ABUW_1972 | *adeS* | 0,1150 | 7,01E-01 | adeS, two-component sensor histidine kinase AdeS |
| ABUW_1973 | *adeR* | 0,3497 | 1,46E-01 | adeR, efflux system response regulator transcription |
| ABUW_1338 | *adeL* | 0,2071 | 3,17E-01 | adeL, multidrug efflux transcriptional repressor AdeL |
| ABUW_1731 | *adeN* | 0,7956 | 8,17E-07 | adeN, multidrug efflux transcriptional repressor AdeN |

| ***Acinetobacter baumannii* AMA40** | | | | |
| --- | --- | --- | --- | --- |
| **Iron uptake** | **Gene name** | **Log_2_FC** | **FDR (P-adjusted)** | **Gene Function** |
| F4T99_00775 | *bauA* | -0,2465 | 2,34E-01 | bauA, TonB-dependent ferric acinetobactin receptor |
| F4T99_12055 | *bfnH* | 1,0665 | 2,91E-25 | TonB-dependent receptor |
| F4T99_09760 | *pirA* | -0,3934 | 7,71E-03 | TonB-dependent siderophore receptor |
| F4T99_03330 | *piuA* | 0,5518 | 1,85E-07 | TonB-dependent siderophore receptor |
| F4T99_14440 | *fhuE_1* | 0,5016 | 1,47E-03 | TonB-dependent siderophore receptor |
| F4T99_14195 | *fhuE_2* | 1,2446 | 2,37E-22 | TonB-dependent siderophore receptor |
| F4T99_09640 | *tonB* | 1,3160 | 8,93E-29 | energy transducer TonB |
| **Antibiotic resistance** | | | | |
| F4T99_19820 | *bla*_NDM-1_ | -0,0322 | 8,83E-01 | subclass B1 metallo-beta-lactamase NDM-1 |
| F4T99_07165 | *bla*_OXA51-like_ | 0,2843 | 1,74E-01 | OXA-51 family carbapenem-hydrolyzing class D |
| F4T99_00690 | *bla*_ADC_ | 0,1135 | 6,19E-01 | class C extended-spectrum beta-lactamase ADC-26 |
| F4T99_01215 | *bla*_GES-14_ | 0,5801 | 2,58E-05 | D-alanyl-D-alanine carboxypeptidase PBP6B |
| F4T99_00275 | *pbp1* | 0,3434 | 5,99E-03 | mrcB, penicillin-binding protein 1B |
| F4T99_03535 | *pbp2* | -0,0056 | 9,82E-01 | mrdA, penicillin-binding protein 2 |
| F4T99_05695 | *pbp3* | 0,0919 | 5,35E-01 | ftsI, penicillin-binding protein PBP3 |
| **Efflux pumps** | | | | |
| F4T99_19380 | *ompA* | 0,3279 | 4,18E-01 | OmpA family protein |
| F4T99_01495 | *carO* | 0,4425 | 1,43E-02 | carO, ornithine uptake porin CarO type 3 |
| F4T99_14865 | *emrB* | -0,4504 | 2,09E-02 | DHA2 family efflux MFS transporter permease |
| F4T99_14870 | *emrA* | 0,0122 | 9,56E-01 | EmrA/EmrK family multidrug efflux transporter |
| F4T99_13390 | *adeA* | -1,2395 | 1,37E-14 | adeA, multidrug efflux RND transporter periplasmic |
| F4T99_13385 | *adeB* | -0,9720 | 8,21E-16 | adeB, multidrug efflux RND transporter permease |
| F4T99_13380 | *adeC* | 0,0917 | 7,44E-01 | adeC, multidrug efflux RND transporter outer membrane |
| F4T99_00380 | *adeF* | -0,6322 | 9,96E-02 | adeF, multidrug efflux RND transporter periplasmic |
| F4T99_00385 | *adeG* | -0,9956 | 7,28E-05 | adeG, multidrug efflux RND transporter permease |
| F4T99_00390 | *adeH* | -0,4568 | 1,53E-01 | adeH, multidrug efflux RND transporter outer membrane |
| F4T99_01850 | *adeI* | -0,6423 | 1,92E-09 | adeI, multidrug efflux RND transporter periplasmic |
| F4T99_01855 | *adeJ* | -0,6694 | 1,02E-08 | adeJ, multidrug efflux RND transporter permease |
| F4T99_01860 | *adeK* | -0,5719 | 1,63E-07 | adeK, multidrug efflux RND transporter outer membrane |
| F4T99_13400 | *adeS* | -0,1764 | 5,44E-01 | adeS, two-component sensor histidine kinase AdeS |
| F4T99_13395 | *adeR* | -0,8913 | 2,98E-05 | adeR, efflux system response regulator transcription |
| F4T99_00375 | *adeL* | 0,2064 | 3,09E-01 | adeL, multidrug efflux transcriptional repressor AdeL |
| F4T99_10390 | *adeN* | 0,4732 | 5,79E-04 | adeN, multidrug efflux transcriptional repressor AdeN |
