# Supplementary material for: Acinetobacter baumannii response to cefiderocol challenge in human urine
Source: Sci Rep. 2022 May 24;12:8763. doi: 10.1038/s41598-022-12829-7 (PMC9128776; doi:10.1038/s41598-022-12829-7)
Supplement: Supplementary file 4 — Supplementary Table S3. [file 41598_2022_12829_MOESM4_ESM.docx]

**Supplementary Table S3:** Minimal Inhibitory Concentrations (MICs) of cefiderocol (CFDC) for 8 Carbapenem-resistant *Acinetobacter baumanii* representative strains and E. coli 1705 performed using CFDC MTS strips (Liofilchem S.r.l., Italy) on Iron-depleted CAMHA (Cation Adjusted Mueller Hinton Agar).

| MICs E-test (mg/L) | | |
| --- | --- | --- |
| Strain | MH | MH 50% HU |
| AB0057 | 0.5 (S) | 0.50 (S) |
| ABUH702 | 1.5 (S) | 1.5 (S) |
| AMA4 | 12* (>256) (R) | 96* (>256) (R) |
| AMA12 | 1.5 (S) | 2 (S) |
| AMA13 | 16* (>256) (R) | 16* (>256) (R) |
| AMA16 | 12* (>256) (R) | 128* (>256) (R) |
| AMA41 | 1.5 (S) | 3 (S) |
| AYE | 24 (R) | 12 (I) |
| *E. coli* 1705 | <0.016 (S) | <0.016 (S) |

* Intra-colonies are present. S: Susceptible, I: Intermediate, R: Resistant

HU: Human Urine. *A. baumannii* cells were cultured in MH, MH supplemented with HU 25%, 50% or 100% HU.
