# Supplementary material for: Acinetobacter baumannii response to cefiderocol challenge in human urine
Source: Sci Rep. 2022 May 24;12:8763. doi: 10.1038/s41598-022-12829-7 (PMC9128776; doi:10.1038/s41598-022-12829-7)
Supplement: Supplementary file 5 — Supplementary Table S4. [file 41598_2022_12829_MOESM5_ESM.docx]

**Supplementary Table S4:** Minimal Inhibitory Concentrations (MICs) of cefiderocol (CFDC) for Carbapenem-resistant *Acinetobacter baumanii* AB5075 and AMA40 strains after 48 and 72 h exposure in HU, performed using CFDC MTS strips (Liofilchem S.r.l., Italy) on Iron-depleted CAMHA (Cation Adjusted Mueller Hinton Agar).

| MICs E-test (mg/L) | | | | |
| --- | --- | --- | --- | --- |
| Strain | MH (48 h) | MH 50% HU (48 h) | MH (72 h) | MH 50% HU (72 h) |
| AB5075 | 0.5 (S) | 0.50 (S) | 0.5 (S) | 0.5 (S) |
| AMA40 | 0.75 (S) | 1.0 (S) | 1.5 (S) | 1.5 (S) |

S: Susceptible, I: Intermediate, R: Resistant

HU: Human Urine. *A. baumannii* cells were cultured in MH or MH supplemented with HU 50%.
